# A successful chronic care program in Al Ain-United Arab Emirates

**DOI:** 10.1186/1472-6963-10-47

**Published:** 2010-02-22

**Authors:** Latifa M Baynouna, Amal I Shamsan, Tahira A Ali, Lolowa A Al Mukini, Moza H Al Kuwiti, Thuraya A Al Ameri, Nico JD Nagelkerke, Ahmad M Abusamak, Nader M Ahmed, Sanaa M Zein Al Deen, Tariq M Jaber, Abdulkarim M Elkhalid, Anthony D Revel, Alhusini I Al Husaini, Fouad A Nour, Hayat O Ahmad, Mohammad K Nazirudeen, Rowaya Al Dhahiri, Yahya O Zain Al Abdeen, Aziza O Omar

**Affiliations:** 1 Ambulatory Health Care Services, SEHA, Al Ain, Abu Dhabi, United Arab Emirates; 2 Family Medicine Institute, Al Ain Hospital, SEHA, Al Ain, Abu Dhabi, United Arab Emirates; 3 Community Medicine Department, Faculty of Medicine and Health Sciences, UAE University, Al Ain, Abu Dhabi, United Arab Emirates; 4 Al Ain Primary Health Care, Al Ain, Abu Dhabi, United Arab Emirates

## Abstract

**Background:**

The cost effective provision of quality care for chronic diseases is a major challenge for health care systems. We describe a project to improve the care of patients with the highly prevalent disorders of diabetes and hypertension, conducted in one of the major cities of the United Arab Emirates.

**Settings and Methods:**

The project, using the principles of quality assurance cycles, was conducted in 4 stages.

The assessment stage consisted of a community survey and an audit of the health care system, with particular emphasis on chronic disease care. The information gleaned from this stage provided feedback to the staff of participating health centers. In the second stage, deficiencies in health care were identified and interventions were developed for improvements, including topics for continuing professional development.

In the third stage, these strategies were piloted in a single health centre for one year and the outcomes evaluated. In the still ongoing fourth stage, the project was rolled out to all the health centers in the area, with continuing evaluation. The intervention consisted of changes to establish a structured care model based on the predicted needs of this group of patients utilizing dedicated chronic disease clinics inside the existing primary health care system. These clinics incorporated decision-making tools, including evidence-based guidelines, patient education and ongoing professional education.

**Results:**

The intervention was successfully implemented in all the health centers. The health care quality indicators that showed the greatest improvement were the documentation of patient history (e.g. smoking status and physical activity); improvement in recording physical signs (e.g. body mass index (BMI)); and an improvement in the requesting of appropriate investigations, such as HbA1c and microalbuminurea. There was also improvement in those parameters reflecting outcomes of care, which included HbA1c, blood pressure and lipid profiles. Indicators related to lifestyle changes, such as smoking cessation and BMI, failed to improve.

**Conclusion:**

Chronic disease care is a joint commitment by health care providers and patients. This combined approach proved successful in most areas of the project, but the area of patient self management requires further improvement.

## Background

Chronic diseases constitute a major global health burden. In the United Arab Emirates one in four people has diabetes, and disorders such as hypertension, asthma, and dyslipidemia are highly prevalent even in early adulthood [1,2]. The quality of care that these patients receive has a tremendous impact on their prognosis and quality of life.

It is well recognized that tight control of hypertension, glycemia and cholesterol are all effective methods of reducing morbidity and mortality [3-5]. In addition there is strong evidence of a positive synergy between these interventions [6], so comprehensive care provided by a single care provider should be the most efficient.

In recent years the role of Primary Health Care has been emphasized as the most cost-effective form of care utilizing standardized pharmacologic regimens that involve a limited number of widely used and relatively nontoxic agents [7]. Standards of care for various chronic diseases have been defined in the context of simple evidence based clinical practice guidelines. Nevertheless, many reports have shown that adherence to standards, despite their simplicity, has often remained low. Training of health care professionals, while essential, is only one part of the solution and would have only a limited impact unless the health system is designed to facilitate the delivery of quality care.

To improve the quality of care, its structure, process, and outcome must all be targeted [8]. It is the design of the care system that determines chronic care quality [7], thus the most successful initiatives are those focusing on the health care system as a whole, such as the Chronic Disease Model which has been developed and implemented in many parts of the world [9-15].

In Al Ain, in 2003, a primary health care project to improve the quality of care provided to patients with diabetes and hypertension was initiated. Its three main objectives were to optimize resource utilization, to improve the management and outcome of care for patients with diabetes and hypertension based on evidence based guidelines, and to improve Health Care Professionals' knowledge and adherence to up-to-date evidence based clinical practice guidelines and thereby improves patients' quality of life and satisfaction.

## Settings and Methods

### Settings

Al Ain is the fourth largest city in the United Arab Emirates with a population of around 400,000 people, of which a quarter are United Arab Emirates (UAE) nationals and the rest expatriates. Since the early 1980s Al Ain primary health care department has operated eleven centrally managed health care centers within the city as well as eight smaller rural centers that serve the periphery of Al Ain. These centers provide highly accessible services to UAE nationals without the need for appointments. As they are free to Nationals and also are connected to the two main hospitals in the city by a good referral system, they are generally preferred to private clinics, especially for chronic conditions. Services at these centres are provided from 8 am to 11 pm during regular week days by staff working in two shifts; with some centers also providing weekend services. These health centers cater for routine ambulatory care and emergency services as well as vaccination, antenatal care and the primary care of chronic disease.

With a single body providing all primary care, Al Ain's primary health care indicators for many areas, such as vaccination are good (coverage 97%). Nevertheless, rapid population growth puts pressure on service provision and concerns were raised with regards to the cost-effectiveness of the long opening hours and poor control of patient flow in the current walk-in system. Health care staff complain of insufficient time per patient during peak hours, especially for those needing more focused care such as diabetics, asthmatics, hypertensives or antenatal patients. To improve this situation a special program was initiated in all 10 urban health care centers - the subject of this study.

Most Health Care Professionals working in these centers are non UAE nationals who have been working in their center for more than 5 years. Few have postgraduate degrees in family medicine or any other medical specialty.

## Methods

Table (1) shows an overview of the project which consisted of multiple interventions with different strategies to be implemented in four stages.

**Table 1:** The major components of the project, including organizational interventions and interventions targeting both patients and health care professionals

Stage	Intervention	Details/Strategies	Aim of intervention
**I. Assessment**	**Flow Audit**	Snapshot of 1-3 days in all centers over all hours covered and of all services	To study patient service mismatch
	
	**Prevalence Study**	Prevalence of conventional CVD risk factors assessed	Quantify problem in community served
	
	**Care of Chronic Disease Audit**	Chart audit of care of DM & HTN	Determine baseline measures of process and outcome of care for the population studied

**II. Evolving Intervention**	**Audit Feedback**	Presentation of the audit results with document of audit summary distributed in a CME presenting recommended care as well.	Stat current practice for the HCP for awareness and reflection and to facilitate uptake of change
	
	**Educational Meetings**	Ongoing educational activities through CME/CNE/workshops for doctors and nurses that focused on the different aspects of the project	Venue to disseminate audit feedback and guidelines
	
	**Piloting**	Tailored intervention piloted in one of the centers and regularly audited including repeat of patient flow study	Trial of the intervention on small scale that can be monitored and adjusted easily and further to use it as a successful example to facilitate change of other centers
	
	**Administration**	Leadership commitment Multidisciplinary participation	To ensure commitment, support and ongoing follow up.
			
		Overall coordinator assigned	
			
		Facilitators for the different tasks	

**III. Intervention**	**Decision Making Aids and Tools**	Follow-up sheets in the chart (colour coded) with reminders of recommended standard of care	To ensure adherence by reminders during consultation and decrease variability
		
		Clinical Practice Guidelines distributed	To ensure implementing evidence based practice and decrease variability
	
**"The structured Care"**		Daily appointment based clinics for DM and HTN patients	To provide protected time for the doctor and patients in clinics preset according to recommended care.
		
	**System Change**	Open access to laboratory and drug formulary	To support and facilitate adherence
		
		Calling reminder system of appointments.	To increase show rate in clinics
		
		Accessibility daily to lab at the point of care in all centers	To support and facilitate adherence
		
	**Information**	Implementing diabetic and hypertensive Evidence-Based Guidelines through the work of the local Clinical Practice Guidelines Working Group	To ensure implementing evidence-based practice and decrease variability. The guidelines adapted by local group giving the ownership to the documents.
	
	**Educational Support**	Educational activities through CME/CNE/workshops for doctors and nurses	To introduce the project tools as guidelines and compare them to the feedback from their practice. Also to cover areas needing increased awareness.
	
	**Self-Management**	Hand held booklet with the patient essential data as agreed on targets for important measures and latest tests result and changes in medications	To empower the patient to be active in the management of his illness.
			
		Health Education Facilitator: Health educationist started weekly visits supervising staff involved in the clinics and to emphasis on Self-Management issues	
			
		Issuing of free blood glucose monitoring devices for home monitoring	
			
		Introducing health education forms	

**IV. Maintenance and Intervention review**	**Audit & Feedback**	Regular Audits with at least one major audit covering all centers yearly	To monitor progress and give feedback to the centers
	
	**HCP feedback**	Continuous communication between implementation team and the HCP in the centers	To ensure compliance and solve any emerging problems
	
	**Patient Feedback**	During visits and satisfaction questionnaire	Patient feedback is important measure

Stage I: Included baseline studies on: i) patient flow system in the centers, ii) care of diabetes and hypertension (in 2004) and iii) a community based study carried out in parallel by the same team as ii) with the objective of assessing the prevalence of cardiovascular risk factors in the Al-Ain population. These were generally perceived to be high, but exact data was lacking despite the clear need for this information. Results from this community survey, which was conducted in 2004-2005, also in Al-Ain, have been published elsewhere [1,16].

The flow study (audit) was done in 2003 in all centers. The audit form included information on patient demographics, date/time of attendance, waiting time, reason for and duration of consultation and details of investigations, prescribing, referral and follow-up. After piloting this form all centers were informed about the findings of the audit and given enough time to become familiar with the form. The number of audit days required per center depended on the size of the center and the services provided.

Centers that provided on-call services at weekends were also required to include a weekend. On audit days each patient utilising any service was allocated a form that was completed by all designated staff members including clerk, nurse, and the General Practitioner. Forms were then collated for data entry and analysis. In total 4947 patients were included in the audit. For logistical reasons the flow audit was only repeated after the intervention in the center where the intervention was piloted.

The baseline diabetes and hypertension care audit was conducted in 2004 in all centers. It was repeated annually in all intervention centers during the intervention. Stage II: Basically this stage followed the **P**lan, **D**o, **S**tudy, **A**ct methodology; where the **Plan **of the intervention was developed locally by each center's management and implemented by a local team in order to instill a sense of ownership of the project. An overall coordinator (LMB) facilitated the coordination and the running of the project.

In **Do **the intervention evolved from communicating the baseline audit results prior to inaugurating changes in settings or resources. In addition a one day workshop for health professionals, on cardiovascular risk factors was conducted at the end of 2004 highlighting assessment and management of risk by means of the Framingham risk score.

In 2005 an intervention was designed on the basis of the responses from the participating Heath Care Professionals (HCP) to the findings of the audit, including the various educational activities following the audit. The major component of this intervention was the establishment of dedicated clinics for chronic conditions, each being tailored to the local situation where chronic patients had been seen in acute care clinics and had received insufficient attention. Other components of the intervention are shown in Table 1. The intervention was initially piloted in the academic health center where medical students and family medicine residents receive their training. All tools used in the project, such as forms, registers, policies and guidelines were piloted, tested and improved in this phase.

The additional intervention consisted of facilitating system change, developing decision making aids, educational activities, and a self-management program. The facilitators had well defined roles and were rotating periodically to review the system of implementation depending on the perceived needs or requests of individual health centers. One facilitator (HA&TA) in charge of health education visited the health centers on a fortnightly basis to monitor patient flow and educate the nurses on various aspects of care including the measurement of waist circumference and the calculation of BMI. She also helped the nurses with patient education on such topics as dietetics and foot care. All patients were provided with "self management cards" which they were encouraged to take to each consultation. Another facilitator (SZ) monitored the overall system including the booking of patients and appointment attendance rates. The medical record facilitator (SZ&FA) reviewed the charts for the forms and the chronic diseases register, and conducted the patient satisfaction surveys.

Decision making support tools were provided including color coded forms (green for diabetes, pink for hypertension and light green for patients with diabetes and hypertension). These included reminders of care guidelines. The Clinical Practice Guidelines were reviewed and adapted by a local multidisciplinary guidelines group comprising general practitioners, family physicians, nurses and pharmacists, from the centers. Guidelines were modified from ATP III [17] for dyslipidemia, ADA [18] guideline,

ICSI and NICE [19] for Diabetes and NICE [20], JNC 7 [21] and European Hypertension society guideline for hypertension [22]. Also the SNAP [23] guideline was included for therapeutic life style changes. The final draft of these modified guidelines was reviewed by a different group, similarly selected from participating centers. The final guideline was introduced in local Continuing Medical Education (CME) activities by the Guideline Group and HCP were encouraged to consult guideline group members for clarifications.

In addition, workshops for nurses and many CME sessions for doctors were devoted to Diabetes, Hypertension, dyslipidemias, and the assessment and management of cardiovascular risk.

In order to address the problems with time allotment, daily appointment based clinics were instigated in all larger centers to manage patients with diabetes and hypertension.

They were staffed by dedicated nurse and doctor.

In the medium sized centers, with lower patient flow, patients were seen in-between other patients, but with appointments distributed throughout the days of the week for better time management. A telephone visit reminder system was introduced in order to decrease the non-attendance rate.

Over the same period access was improved to both drugs and laboratory investigations.

Centers without their own laboratory were provided with phlebotomist services to obviate the need for referring patients to the central laboratory. Finally, self management was promoted by means of hand held booklets that included all relevant patient data, space for communication with hospital based specialists, as well as self-management measures agreed upon between doctor and patient. Diabetes patients were issued free blood glucose monitoring devices for home monitoring.

Stage III: After the success of the intervention in the pilot center the intervention was expanded to all other city centers. One of the elements learned in the pilot phase and implemented in all centers was how to spread all recommended care over several visits and how to use this to adjust medication. Nurses were involved in ensuring that required investigations were performed prior to the consultation

Stage IV: Maintenance and intervention review. Since the initial intervention an annual audit of diabetes and hypertension care has been conducted in all centers.

### Methods of evaluation

Changes in standards of care were tracked by chart audits.

The first audit in July 2004 perused 672 files, then after the implementation of the project in the pilot center in 2005 164 files were audited. The audit checklist included information on the process and outcome of care as per guideline recommendations, such as treatments prescribed, documentation of family history, smoking and physical activity, recording of blood pressure, BMI and waist circumference in physical examination and investigations ordered - ECG, Creatinine, HbA1C, lipid profile, and TSH. The auditing was conducted by specially selected nurses temporarily made available by participating health centers.

In 2007 information was extracted from 1402 files. For all patients measures were recorded both before (2006) and after (2007) the intervention. This was repeated in 2008 (715 files). In 2008 three centers were not audited for organizational reasons, and the pilot center was neither audited in 2006 nor in 2008. In order to determine the effect of the intervention the 2008 audit included the same patients audited in 2007, as well as a sample of new patients for comparison. The number of records audited was determined by the size of the center. For large centers a total of around 100, and for medium size centers 60-80 patients was targeted. The attendances for large, medium and small centers are 250-300, 150-200 and 100-150 patients per day respectively.

The chronic disease clinics attracted around 3000 patients per month in the ten centers. Data are not available to determine how many diabetic or hypertensive patients are registered at each center. Although registers are available and each patient has a unique "disease specific identifier" (e.g DM91) in these registers, we still found deficiencies and duplications in the registers. In order to avoid problem with sampling from these imperfect registries, a sample was extracted instead from the appointment books of patients attending in the three month period prior to the audit.

Satisfaction of patients about the project was sought through annual interviews. Staff satisfaction was monitored by a constant feedback from the health care professionals to the implementation team. Comments on feasibility and problems associated with the project were elicited during face-to-face interviews by facilitators during regular (more than once weekly) visits to the centres.

### Analytical methods

To compare indicators before and after implementation, standard paired and independent sample t-tests were used where appropriate. SPSS (v 15) was used for all comparisons.

## Results

**Figure 1:**
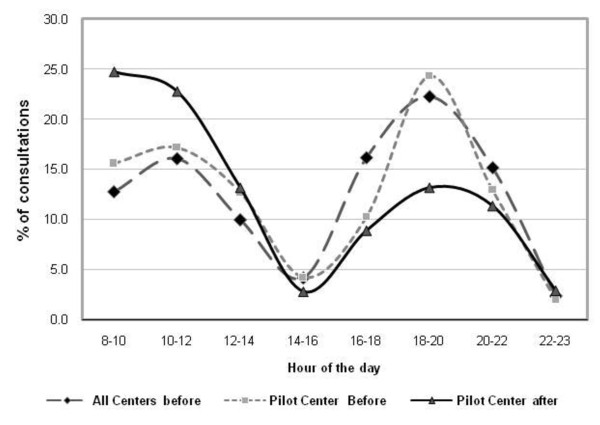
**Flow of patients attending before the intervention in the pilot center and all other 19 (10 urban health centers, 1 labor clinic, 8 rural clinics) centers (over 3 days of the flow audit) and after the intervention in the pilot center (over a whole year)**.

In the patient flow analysis, an obvious mismatch of demands and supply of services was detected (Figure (1)). Despite a peak demand between 16:00 to 18:00 daily, staff availability was constant during all 16 opening hours from 8.00 to 23:00. The population characteristics did not show a difference between the study periods with regards to age, sex, duration of disease, or other variables. Diabetes and hypertension on the other hand constitutes almost 13% and 12% of the total number of visits to the centers included in the project in 2004 and 2007 respectively as shown in Table 2 and 3.

**Table 2:** Characteristics of the patients' population before and after intervention.

	2004 (before) Number (%)			2007 (after) Number (%)		
	**DM**	**HTN**	**DM & HTN**	**DM**	**HTN**	**DM & HTN**

**Male**	72 (51.1)	77 (47.8)	207 (55.5)	193 (50.5)	178 (38.9)	184 (43.5)

**Female**	69 (48.9)	84 (52.2)	166 (44.5)	189 (49.5)	280 (61.1)	239(56.5)

**Age (years)**						

<= 25	2 (1.4)	2 (1.3)	3 (0.8)	2 (0.5)	7 (1.5)	0 (0)

26-35	6(4.3)	2 (1.3)	3 (0.8)	9 (2.4)	10 (2.2)	5 (1.2)

36-45	20 (14.3)	16 (10.1)	41 (11)	64 (16.8)	62 (13.6)	35 (8.3)

46-55	57 (40.7)	50 (31.6)	114 (30.6)	133 (35)	142 (31.1)	133 (31.4)

56-65	31 (22.1)	38 (24.1)	126 (33.9)	106 (27.9)	119 (26.0)	135 (31.9)

66-75	18 (12.9)	39 (24.7)	70 (18.8)	41 (10.8)	74 (16.2)	76 (18.0)

>75	6 (4.3)	11 (7)	15 (4)	25 (6.6)	43 (9.4)	39 (9.2)

**Nationality**						

UAE	125 (88.7)	155 (96.3)	351 (94.1)	358 (93.8)	425 (92.8)	392 (92.7)

Non-UAE	16 (11.3)	6 (3.7)	22 (5.9)	24 (6.2)	33 (7.2)	31 (7.3)

**DM duration **min/max (mode)	1/17 (1)			1/32 (3)		

**HTN duration **min/max (mode)	1/20 (5)			1/32 (3)		

* Patients with (DM) only, ** Patients with hypertension only (HTN), *** Patients with both conditions (DM&HTN).

**Table 3:** Total number of visits of all patients and patients with DM and HTN to the Primary Health Care (PHC) centers participating in the project in 2004 and 2007.

2007						2004						
**Total DM&HTN**	**%**	**DM visits**	**%**	**HTN visits**	**No. of visits**	**Total DM&HTN**	**%**	**DM visits**	**%**	**HTN visits**	**No. of visits**	**PHC Centers**

7.9	3.7	2140	4.2	2453	57776	14.2	6.1	1975	8.2	2658	32611	**Masoudi**

15.2	6.5	2047	8.8	2777	31683	15.1	6	2021	9	3029	33505	**OTC**

13.8	6.4	2992	7.4	3431	46449	17.3	7.5	3613	9.8	4687	48054	**Kabisi**

13	5.5	1835	7.5	2510	33509	16.5	7.3	1972	9.2	2507	27148	**Hilli**

14.5	6	1511	8.6	2173	25391	14	5.7	1851	8.4	2733	32683	**Niyadat**

11.3	4.8	1150	6.5	1569	23976	11.4	4.6	1285	6.8	1903	28043	**Mueiji**

8.8	4.4	3626	4.4	3584	81991	12.3	6.4	3888	5.9	3559	60631	**Mezyad**

12	6	3320	5.9	3252	54915	11.5	5.6	3561	5.9	3740	63675	**Maqam**

9.5	4.5	725	5	817	16187	9.3	4.7	1195	4.6	1177	25504	**Zakher**

11.7	6.8	2952	4.8	2096	43252	10.1	5.5	3076	4.6	2608	56394	**Yahar**

**11.8**	**5.5**		**6.3**			**13.2**	**5.9**		**7.2**			**Average (%)**

HTN Hypertension, DM diabetes mellitus.

In the parallel prevalence study [1] a prevalence of diabetes and hypertension of 23.3% and 21% respectively was found. Despite this high prevalence, the baseline audit of process and outcome of care for these conditions revealed an alarming situation (Table 4).

**Table 4:** Adherence to recommended care in process measures broken down by the four time periods.

	2004 (before intervention)		2006* (after audit feedback and educational activities)		2007 (intervention complete, including structured care)		2008 (follow-up)	
**Measure assessed**	**No.**	**%**	**No.**	**%**	**No.**	**%**	**No.**	**%**

Smoking last visit	541	80.5	1133	89.7	1309	93.4	638	89.2

Physical Activity	7	1.0	797	63.1	1339	95.5	651	91.0

BMI	15	2.2	842	66.7	1334	95.1	624	83.5

Systolic blood pressure	665	97.2	1137	90.0	1395	99.5	657	91.9

Diastolic blood pressure	665	97.2	1137	90.0	1395	99.5	657	91.9

Urine R/E	174	25.0	590	46.7	850	60.6	591	82.7

Microalbuminurea	0	0	533	42.2	1105	78.8	598	83.6

Creatinine	88	13.0	846	67.0	1263	90.1	NA	NA

HbA1c (in DM)	115	22.9	411	51.1	785	89.4	349	76.0

Total cholesterol	323	47.7	836	66.7	1268	90.4	574	80.3

LDL	74	10.9	555	56.1	1247	88.9	569	79.6

HDL	49	7.2	693	54.9	1264	90.2	572	80.0

TG	321	47.4	834	66.0	1256	89.6	574	80.3

Ophthalmology referral	37	5.5	278	22.0	712	50.8	224	31.3

*Pilot center excluded, NA not assessed

Outcome: With the structured care intervention piloted in 2005-2006 measures of quality of care were constantly improving. This started with a correction in the management of patient flows. This was improved by moving patients with identified specialized needs of care such as antenatal care, well baby care, and chronic diseases to special, appointments based, clinics (Figure 1). Thus, after this change, in the pilot center, 24% of consultations were for appointment in the specialized clinics, which 77.4% of the patients attended for more than 6 months and 65.2% for a whole year. This flow management obviated the need for hiring additional staff, in fact hiring was frozen for the period of the project and several staff had even left as a result of retirement or moving jobs.

In the ongoing post-intervention audits indicators of process and outcome of care showed significant improvements (Tables, 4, 5, 6 and 7), including documentation of patients' history, examinations and investigations (Table 4). This notably included recording of smoking status and blood pressure, irrespective of reason for attendance.

**Table 5:** The change outcome measures over the 4 time-periods.

	2004 (before intervention)	2006* (after audit feedback and educational activities)	2007 (intervention complete, including structured care)	2008 (follow-up)
	**%**	**%**	**%**	**%**

Smoking last visit (males)	16.4	13.4	12.7	13.2

Physical Activity	70.0	56.8	62.7	42.7

BMI (% <30)	31.2	56.5	54.8	58.1

Blood Pressure control in HTN				

% Blood pressure <= 140/90	57.0	51.1	72.9	67.5

Blood Pressure control in DM				

% Blood pressure <= 130/80	31.0	32.4	51.6	42.9

HbA1c (% < 7) in DM	18.3	41.4	43.1	45.6

HbA1c (% < 9) in DM	47.0	69.5	77.7	80.0

HbA1c (% < 10) in DM	61.7	79.6	88.3	90.0

Total cholesterol (<200)	25.0	50.8	73.1	79.6

LDL (<100 in DM)	21.1	26.9	34.9	39.4

LDL (<130 in HTN)	10.0	40.4	60.6	59.0

HDL (>40)	48.9	56.3	54.4	20.0

TG (<150)	25.8	67.1	77.3	81.3

**Table 6:** Paired t-test of outcome measures comparing before and after the intervention.

	(2006 mean-2007mean)*	No.	P value	(2007 Mean-2008 Mean)**	No.	P value
**Hypertension**						

**BMI**	29.8-29.9	675	**0.81**	29.2-29.6	421	**0.4**

**HbA1c in DM**	8.0-7.3	265	**<0.001**	7.19-7.17	218	**0.8**

**Total cholesterol**	200.7-184.6	634	**<0.001**	185.8-162.3	408	**<0.001**

**LDL**	130.9-118.8	538	**<0.001**	119.0-104.9	403	**<0.001**

**HDL**	43.5-43.46	537	**0.9**	44.0-40.2	410	**0.07**

**TG**	138.9-122.2	624	**<0.001**	125-105.6	406	**<0.001**

**Diabetes Mellitus**						

**BMI**	29.2-29.4	572	**0.015**	28.5-29.2	383	**0.015**

**HbA1c in DM**	8.4-7.7	436	**<0.001**	7.8-7.6	321	**0.035**

**Total cholesterol**	197.8-180.9	557	**<0.001**	184.1-155.0	370	**<0.001**

**LDL**	126.5-113.5	478	**<0.001**	115-98.1	366	**<0.001**

**HDL**	42.9-43.0	478	**0.8**	44.1-40.4	370	**0.019**

**TG**	148.2-130	552	**<0.001**	131.86-107.6	369	**<0.001**

**Blood pressure control**						

**In patients with DM**						

**Systolic BP**	125.4-122.2	179	**<0.001**	122.2-122.3	189	**0.13**

**Diastolic BP**	79.8-76.6	179	**<0.001**	76.7-75.3	189	**0.01**

**In patients with HTN**						

**Systolic BP**	138.9-132.8	414	**<0.001**	133.2-131.6	226	**0.07**

**Diastolic BP**	85.8-81.3	415	**<0.001**	82.3-79.4	228	**<0.001**

**In patients with HTN and DM**						

**Systolic BP**	137.8 -131.9	389	**<0.001**	133.1-134.7	237	**0.1**

**Diastolic BP**	83.5-79.9	389	**<0.001**	83.4-80.2	237	**0.002**

*2006-2007 indicates the change in 2007 compared to 2006

** 2007-2008 indicate the change in 2008 compared to 2007

**Table 7:** The change in prescribing diabetic medications in diabetics and blood pressure lowering medications in hypertensives in the 4 time-periods.

	2004 (before intervention)		2006 (after audit feedback and educational activities)		2007 (Intervention complete, including structured care)		2008 (follow-up)	
	**No.**	**%**	**No.**	**%**	**No.**	**%**	**No.**	**%**

**Aspirin**	212	**31.3**	412	**32.6**	834	**59.5**	481	**67.3**

**Statin**	204	**30.1**	388	**30.7**	621	**44.3**	427	**59.7**

**Other LLM**	NA	NA	NA	NA	146	**10.4**	82	**11.5**

**DM Rx**								

**No medication**	67	**13.3**	82	**20.6**	25	**6.1**	25	**6.1**

**1 medication**	255	**50.5**	133	**33.4**	104	**25.4**	78	**19.0**

**2 medication**	174	**34.5**	140	**35.2**	194	**47.4**	198	**48.3**

**3 medication**	8	**1.5**	42	**10.6**	77	**18.8**	90	**22**

**4 medication**	0	**0**	1	**0.3**	9	**2.2**	19	**4.6**

**On Insulin**	NA	**NA**	NA	**NA**	47	**5.8**	18	**4.5**

**HTN Rx**								

**No medication**	57	**10.7**	101	**20.2**	33	**7.3**	20	**2.2**

**1 medication**	28	**52.8**	196	**39.3**	174	**38.4**	163	**16.5**

**2 medication**	156	**29.3**	116	**23.2**	185	**40.8**	187	**41.1**

**3 medication**	32	**6.0**	28	**5.6**	57	**12.6**	75	**35.8**

**4 medication**	6	**1.1**	3	**0.6**	4	**0.9**	10	**4.4**

NA, not assessed

Also, the recording of BMI improved substantially from 2.2% in 2004 to 95.1% in 2007. Most likely only apparently obese patients had their BMI recorded in 2004 as around 70% of them had BMIs above 30 kg/m2.

Adherence to Clinical Practice Guidelines adopted by the project, (Table 4) regarding recommended investigations such as kidney function tests, HbA1c and lipid profiles, clearly improved. In 2004 these were available for very few patients but this improved in 2006-2007 following the structured care intervention, and permission given (in 2005) to General Practitioners to order LDL and HDL tests.

With regards to outcome, neither smoking cessation nor BMI were much affected by the intervention. Smoking dropped from 16.4% in 2004 to around 13% in subsequent years while obesity was consistently prevalent in approximately 50% of patients (Table 5, Table 6).

In contrast, measures of disease control in diabetes and hypertension improved. Blood pressure control was impressive and significantly improved with around two third of patients meeting target values of 140/90 or less in, 2007 and 2008 (Table 5, Table 6).

HbA1c in diabetics had dropped significantly with 80% having values below 9 in 2008 compared to 61.7% in 2004 and 69.5% in 2006. The median dropped from 8 in 2006 to 7.5 in 2007 and to 7.2 in 2008 which was significant by paired t-test, (Table 5, Table 6). Systolic blood pressures in diabetics dropped significantly in 2007, from an average of 125.4 in 2006 to 122.2 in 2007 and remained the same in 2008. Diastolic blood pressure on the other hand, kept improving over years, (Table 6). Nevertheless, around half the diabetics met the recommended target of 130/80 in 2007 compared to one third in 2006 and 2004, (Table 5).

**Figure 2:**
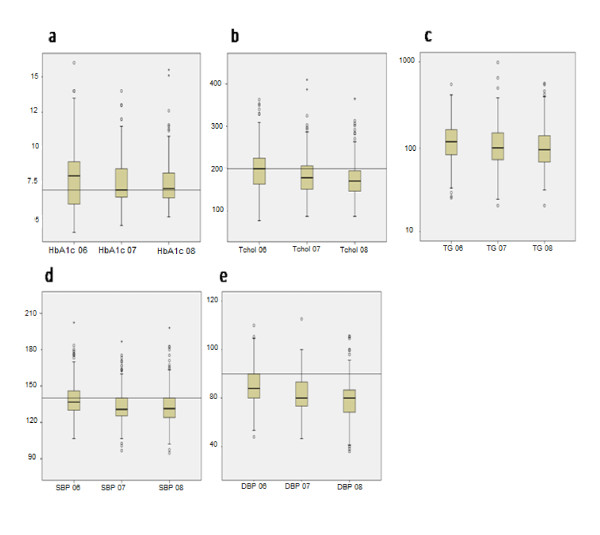
**The change in the main outcome measures during the years of the project**. **a**. HbA1c, **b**. Total Cholesterol, **c**. Triglycerides, **d**. Systolic Blood Pressure (SBP), **e**. Diastolic Blood Pressure (DBP).

Lipid profiles showed a significant improvement in total cholesterol, LDL and triglycerides, since more than three quarter of patients met target values in 2007 and 2008 compared to only a third in 2004 and a half in 2006 (Table 5). However, this was not the case for HDL which remained the same or even significantly dropped in the case of diabetics in 2008. Figure 2 shows the changes in all these indicators. These changes in disease control measures reflected significant increases in prescriptions of aspirin and statins by General practitioners as shown in Table 7. Prescribing of aspirin and lipid modifying agents increased from one third of patients in 2004 and 2006 to two thirds in the structured care intervention years in case of aspirin and to half of the patients in case of statins.

The number of medications per patients also increased over the intervention years. For example, more than three quarters of diabetes or hypertension patients were on two or more medications in 2008, reflecting current guidelines. Insulin use in diabetics, however, was less than 5% in both 2007 and 2008.

Ophthalmology referrals markedly increased from 5.5% in 2004 to 50.8% in 2007 but decreased to 31.3% in 2008. However, other recommended referrals such as dental, nutritional and vaccination for influenza and pneumonia were not audited, since routine referral to these services was impossible due to limited resources and large case loads.

**Figure 3:**
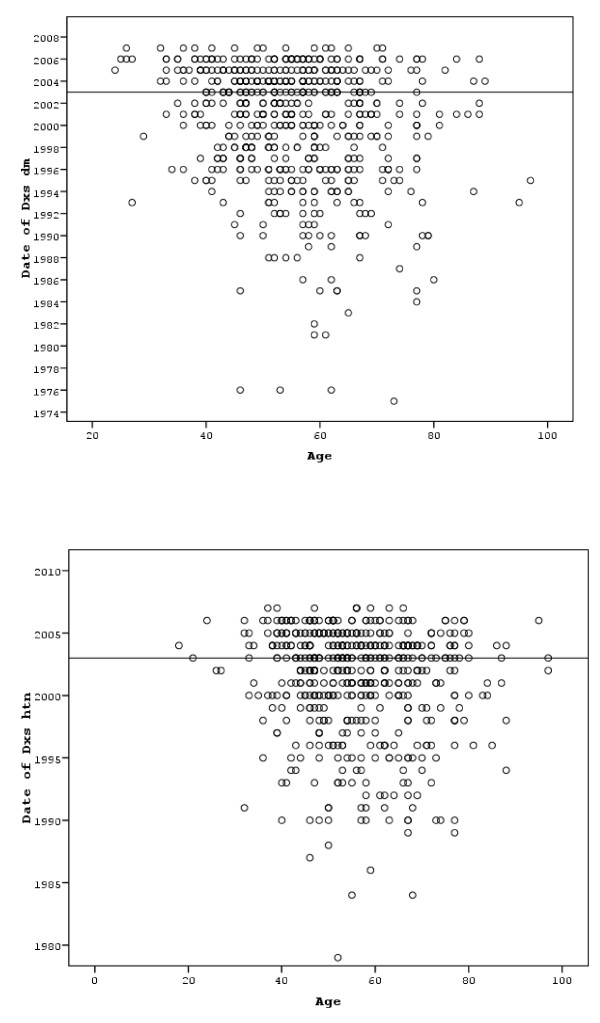
**3-a. The duration of diabetes in the studies population in relation to age, 3-b**. The duration of hypertension in the studied population in relation to age.

In addition, the number of patients diagnosed with diabetes or hypertension increased in recent years as evident from Figure 3-a and 3-b.

## Discussion

Starting with the care audits showing very poor levels of care in 2004 similar to an earlier audit carried out in 1998 [24] and the interventions implemented, the initial audit with feedback coupled with educational activities clearly led to a marked improvement as indicated by a notable change in indicators between 2004 to 2006. These elements were particularly effective in other studies where the baseline adherence to recommended practices was low and the intensity of audits and feedback was high [25].

Nevertheless, most improvements occurred only later with the second structured intervention introduced after 2006 that directly targeted the delivery of services to patients with chronic conditions by introducing special dedicated clinics. The success of structured intervention makes it clear that the lack of knowledge of health care professionals' may be less of a constraint than other structural factors. Improvements seen in our project were comparable to local and international benchmarking figures for good clinical care for both diabetes and hypertension [26-31].

Improvements were achieved in both process and the outcome of care (Table 4, 5 and 6) in response to different elements of the project. Specifically, the change in drug prescriptions (Table 7) came after structured care was introduced, suggesting that the additional resources (time!) available per patient to adjust patients' management plans paid off. Prescribing aspirin and statins had increased steadily, but unfortunately we were unable to assess whether the increase were in accordance with good practice guidelines. Nevertheless, our figures are comparable to other research-based interventions that at best reached 75 and 88% [6]. In contrast, insulin is still prescribed infrequently, <5% in both 2007 and 2008. Whether this is due to doctors' inexperience in using insulin or patients' preferences remains unclear and is an area for future study.

Systolic blood pressure in diabetics remained constant in 2008 after having improved in 2007. In contrast, diastolic blood pressure, HbA1c and most lipid profiles improved with time. Perhaps, doctors were not targeting blood pressure enough in diabetic patients to meet the recommended standards, and may have been more focused on controlling blood sugar and HbA1C. Patients also might have paid insufficient attention to their blood pressure if they are not hypertensive. Nevertheless, as a blood pressure control (particularly systolic blood pressure) is neither 'frequently nor easily obtained' [32], this area may need additional measures especially because lowering blood pressure in diabetics is cost saving [5].

Ophthalmology referrals improved from 5.5% in 2004 to 50.8% in 2007, but decreased to 31.3% in 2008. This decrease may be due to doctors' loss of adherence to recommended care or due to patients' reluctance to accept long hospital waiting times. Better coordination of care with local hospitals in important areas, such as ophthalmology and nephrology, clearly needs improvement.

Unfortunately, our interventions were less successful in redressing life-style related risk factors, such as obesity, physical inactivity and smoking. These domains may need more time and resources, with perhaps a different type of intervention [33]. A deficiency in our project was a lack of nutritionists and health education specialists in the participating centers to reinforce efforts by other HCPs and assess patients' self-management. A recent study suggested that absence of such resources was a major barrier to weight management in Emirati women at risk of DM, as perceived by HCP [33]. Self-management may be another important tool in addressing life-style factors. In our intervention, a hand-held booklet specifying goals in the self management of their illness was distributed to all patients. Also many patients were advised to obtain a home blood pressure monitoring device and a glucometer with strips, which has been freely available since 2007 for diabetic patients. However, the success (or lack of it) of these measures remain to be fully analyzed and understood. For self-management to succeed, both health service support and the acceptance by the patients of their responsibility are crucial. Historically in our culture (and elsewhere), patients often delegated their responsibility in this respect to the doctors they trusted, as this is a meaningful attitude for many other conditions.

The increase in diagnoses of diabetes and hypertension in recent years could be due to various factors, such as a change in cut-off values, intensified screening, as well as a true increase in prevalence. Strategies used in this project, with an organized system of regular follow-up and the use of dedicated clinics [34] significantly improved the care of chronic disease patients [9-15] and may also have improved the case detection rate. Nevertheless, the number of patients treated for diabetes and hypertension in our clinics falls short of the estimated total number of affected individuals, and many patients currently remain untreated, which clearly needs to be addressed in futures interventions.

Collaboration with health care staff working in the centers is key to success. While some aspects of the intervention may be experienced as stressful, we found that many were particularly content with the dedicated clinics, but a full analysis of their experiences remains to be done. Other important elements of this project were chart reminders and educational meetings which were shown to be effective, but only in combination with other strategies [35,36] since educational meetings alone were likely to be effective in changing complex behaviors [37].

Our findings also have relevance for other Gulf countries as many resemble the UAE in their high cardiovascular risk profile as well as their health care system. In addition, our approach may be extended to the management of other chronic conditions. Yet each intervention may need to be piloted separately, as a major limitation of our study was its confinement to a single city in one country, and with a single project management throughout, makings it difficult to generalize to other places and circumstances.

Nevertheless, a major lesson to be learnt from this experience is the value of a single centrally coordinated primary health care system in this city, which facilitated the project and made it a relatively cost effective intervention. This is more obvious if we compare this project to a similar project implemented in the United States targeting the care of chronic diseases on the basis of payment for performance [38]. Financial incentives were necessary in a fragmented primary health care system consisting of isolated different sized practices to implement similar interventions through programs such as Physician Quality R Initiative (PQRI) [39].

Sustainability of the improvements is a major concern. Inevitably, changes in the organization that affect the program occur. Early in 2008 three centers were moved to another managing body and in July 2008 the other centers of the department of Primary Health Care were brought under new administration. "Old" projects such as our intervention were allocated a low priority. Also we lost one of the facilitators. In spite of these two factors, decreased administrative endorsement and loss of the facilitator, the program was successfully maintained by the centers' staff. Yet, Tables 4 &5 demonstrate an overall lack of further improvements and a trend to a decline in some key measures but as in Table 6 none of the decline was significant. And in a more recent audit in 2009, the quality of care was very similar to that found in 2008, which is reassuring for the second year after the change.

We believe that because the project was initiated internally with no external intervention (not ready for the implementation plan and no new people allowed the change) gave a sense of ownership among centers, probably facilitating sustainability.

However, for further improvements, e.g. for diabetics with hypertension, managing nephropathy, self-management of life-style factors, shared care with the hospital, optimizing dyslipidemia management etc., and additional components still to be developed, should be added to the project.

The limitation of this study is mainly its descriptive nature and that it compares after to before the intervention rather than being a factorial randomised controlled study. This makes it difficult to attribute the success of the project to specific components and reduces its external validity.

## Conclusion

We believe that awareness of the problem of diabetes and hypertension in our community, and the clear need to have special organized care for this group of patients in the health care system, may have facilitated the uptake and sustainability of the project. But awareness is not sufficient, since without the other project components - specifically the dedicated personnel and clinics - the intervention would not have been maintained.

## Competing interests

The authors declare that they have no competing interests.

## Authors' contributions

LMB: Coordinated the project and wrote MS, AIS: Member in project working group,

TAA: Member in project working group, LAM: Member in project working group, MHK: Member in project working group, TAA: Project Facilitator and Member in project working group, NJDN: Statistical advice and contributed to MS writing, AMA: Member in project working group, NMA: Member in project working group, SMZ: Project Facilitator and Member in project working group, TMJ: Member in project working group, AME: Member in project working group, ADR: Member in project working group, AIA: Member in project working group, FAN: Member in project working group, HOA: Project Facilitator and Member in project working group, MKN: Member in project working group, RA: Member in project working group, YOZ: Member in project working group, AOO: Project Facilitator and Member in project working group. All Authors have read and approved the final manuscript.

## Pre-publication history

The pre-publication history for this paper can be accessed here:

http://www.biomedcentral.com/1472-6963/10/47/prepub
